# Apple Polyphenol Extract Suppresses *Clostridioides difficile* Infection in a Mouse Model

**DOI:** 10.3390/metabo12111042

**Published:** 2022-10-30

**Authors:** Zhengjie Wu, Qiaomai Xu, Ailing Li, Longxian Lv, Lanjuan Li

**Affiliations:** 1 State Key Laboratory for Diagnosis and Treatment of Infectious Diseases, National Clinical Research Centre for Infectious Diseases, National Medical Center for Infectious Diseases, Collaborative Innovation Centre for Diagnosis and Treatment of Infectious Diseases, The First Affiliated Hospital, Zhejiang University School of Medicine, 79 Qingchun Rd., Hangzhou 310003, China; 2 Jinan Microecological Biomedicine Shandong Laboratory, Jinan 250021, China; 3 Shulan (Hangzhou) Hospital, Hangzhou 310003, China

**Keywords:** polyphenol, *Clostridioides difficile*, intestinal microbiota, metabolism

## Abstract

Fruits such as apples are a dietary source of polyphenols and have health benefits. We studied the benefits of apple polyphenols in reducing intestinal infections. We explored the potential roles of apple polyphenols in combating *Clostridioides difficile*-induced intestinal infections by modulating the intestinal microbiota and metabolism in our study. Mice fed with apple polyphenols exhibited higher survival rates and improved diarrhea symptoms in a *C. difficile* infection mouse model given once-daily apple polyphenol extract (200 or 400 mg/kg bw) or phosphate-buffered saline. Feeding polyphenols enhanced anti-inflammatory effects and colon barrier integrity. In addition, apple polyphenols mitigated intestinal microbiota disorders in *C. difficile* infection, modulating the intestinal microbiota and increasing the abundance of beneficial microbiota. Apple polyphenols also improved fecal metabolic alterations in *C. difficile*-infected mice and modulated the expression of pathways related to intestinal inflammation. Our results suggest that apple polyphenol extract is a potential prebiotic agent that affects the intestinal microbiota and metabolism, thereby positively influencing intestinal infections.

## 1. Introduction

A stable gut microbial community protects against *Clostridioides difficile* [1], but broad-spectrum antibiotics destroy the natural microbiota, allowing pathogens to multiply and causing *C. difficile* infection (CDI) [2]. The infection is associated with dysbiosis of the intestinal microbiota with antibiotic use [3], and there is a loss of multiple short-chain fatty acid (SCFA) producing bacteria in *C. difficile* patients [4].

Diet can affect the composition and function of the gut microbiota and greatly influences the formation of the microbial structure. Prebiotics are indigestible food components that stimulate the growth of beneficial intestinal bacteria, including *Bifidobacteria* and *Lactobacilli*, and thus benefit host health [5]. Prebiotics can influence the composition of the host’s intestinal microbiota and improve inflammation and associated metabolic disorders in the organism. In recent years, with the expansion of relevant research, scientists have discovered that some plant actives can act as prebiotics; they can regulate the host’s intestinal metabolism and microbiota. Phenolic substances and phytochemicals are considered prebiotics with the proposed definition [6]. Dietary polyphenols are secondary metabolites found in foods, such as fruits, vegetables, and tea [7]. They are currently widely studied due to their antioxidant and anti-inflammatory characteristics [8] and are beneficial to commensal bacteria while inhibiting potential pathogens, such as *C. difficile* [9]. Dietary polyphenols can provide indirect protection of its immunomodulatory and anti-inflammatory role by activating endogenous defense systems, such as NF-κB activation and PI3K/Akt pathways [10].

Apple polyphenols are compounds with a variety of health-promoting properties and attracted widespread interest for the health benefits. As a dietary supplement, apple polyphenol extract (APE) has various beneficial effects on humans. Although the composition of the gut microbiota would influence the metabolism of apple polyphenols, apple polyphenols and its metabolites can also alter the diversity and composition of the microbiota. It can protect ulcerative colitis [11] and hepatic steatosis by gut microbiota [12]. The main active constituents of apple polyphenols include phloretin and chlorogenic acid [13]. Apple polyphenols can reduce the oxidative stress and attenuate the inflammatory response by reducing the level of cytokines [14]. APE is safe and has little toxicity at the dietary level [15]. The specific composition and immunomodulatory activity of apple polyphenols are still being studied.

The objective of this study was to explore the interaction mechanism of apple polyphenols and gut microbiota to affect *C. difficile*-induced intestinal infection. In this work, the role and mechanism of APE were assessed using a CDI mouse model. We analyzed the protective effect of different doses of APE against CDI and its effects on the regulation of the inflammatory response, intestinal barrier, transcriptome, and metabolism; in addition, the gut metabolic structure was also assessed by SCFAs. 

## 2. Materials and Methods

### 2.1. Animal Experiments

**Figure 1:**
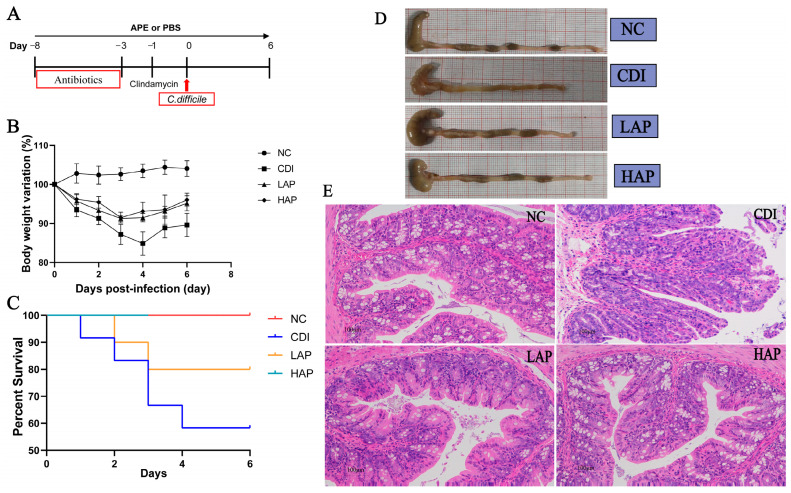
Effects of APE on *C. difficile*-infected mice. (**A**) Experimental design. (**B**) Body weight change. (**C**) Survival curve. (**D**) Appearance of colon. (**E**) H&E staining images of colonic tissues of mice in each group. PBS, phosphate-buffered saline.

APE (purity > 80%) was purchased from JF-Natural (Tianjin, China) [16]. Female C57BL/6 mice were randomly divided: the NC group (N = 8), control uninfected mice; the CDI group (N = 12), infected mice that received pretreatment with antibiotics and *C. difficile* attack on day 0; the LAP group (N = 10), infected mice managed with APE at 200 mg/kg/day; and the HAP group (N = 10) with APE at 400 mg/kg/day. The CDI and NC groups were accordingly administered with phosphate-buffered saline (PBS). As the infection model previously described [17], the five-day antibiotics of kanamycin, gentamicin, colistin, metronidazole, and vancomycin were mixed in daily water (Figure 1A). It was switched to normal water for 2 days and received clindamycin (10 mg/kg body weight, ip) for 1 day. Further infection with 10^8^ colony-forming units of the *C. difficile* strain VPI 10463 (ATCC 43255) was then administered by oral feeding. The body weight and clinical symptoms of mice after infection were detected.

### 2.2. Histopathological Analysis

Colon tissue was fixed in formalin. Sections were stained with hematoxylin and eosin (H&E). The neutrophil and macrophage of the colon tissue were observed using Ly6G and F4/80 immunostaining. The intestinal barrier of the colonic tissue was observed by ZO-1 immunofluorescence staining [18].

### 2.3. Hematological Assays

The serum concentrations of several cytokines were measured using a Bio-Plex Cytokine Panel (Bio-Rad, Hercules, CA, USA). Lipopolysaccharide (LPS) binding protein (LBP) ELISA kit (Abcam, Cambridge, UK) were used to assess serum inflammation [19].

### 2.4. Quantitative Real-Time PCR (RT-qPCR)

Total RNA extraction was carried out using an RNeasy Plus Mini Kit (Qiagen, Valencia, CA, USA). RT-qPCR was detected on a VIIA7 RT-PCR system (Applied Biosystems, Waltham, MA, USA). The mRNA level was normalized to β-actin. The primer information is listed in Supplemental Table S1 [20].

### 2.5. Microbial Community Analysis

Microbiota analysis was performed on fecal samples collected prior to execution at day 6. DNA was extracted from feces using a DNA Kit (Qiagen, Valencia, CA, USA). A universal primer (Supplementary Table S1) was used to amplify the V3–V4 of the 16S rRNA gene. An Illumina NovaSeq platform (Illumina, San Diego, CA, USA) was used for PCR amplification, library preparation, and DNA sequencing [21]. Raw sequences were trimmed, filtered, and then merged. QIIME 2 software was used for amplicon sequence variant assignment, clustering, etc., and subsequent microbial diversity and differential enrichment [22]. The sequencing data were uploaded to the SRA database (PRJNA868051).

### 2.6. Metabolic Analysis

Gas chromatography–mass spectrometry (GC-MS) was used for the metabolic analysis of cecum contents. The processed samples were prepared as previously mentioned [23] and then analyzed using an Agilent 7890B-5977B GC-MS system (Agilent, Santa Clara, CA, USA). Metabolites were identified by NIST and further analyzed.

For the quantitative analysis of SCFAs in cecum contents, the samples were mixed with water, vortexed, centrifuged, and mixed with ethyl acetate. After centrifugation, the supernatant was incubated and then transferred to sample tubes for further analysis [20].

### 2.7. Transcriptome Analysis

Colon RNA quantification, library creation, and sequencing were performed as previously described [21]. The differential expression of genes between groups was analyzed by the R package DESeq2 [24]. The statistical enrichment of Kyoto Encyclopedia of Genes and Genomes (KEGG) pathways was performed based on the differentially expressed genes [25]. The data of RNA sequencing are available at the SRA database (PRJNA868109).

### 2.8. Statistical Analysis

Data are expressed as mean ± SEM. Statistical analyses were performed using ANOVA or Student’s *t* test with SPSS (version 20.0; SPSS, Chicago, IL, USA). Statistical significance was defined by *p* value < 0.05.

## 3. Results

### 3.1. APE Reduces Colitis in C. difficile Infection

To explore the benefits of APE in vivo, we constructed a mouse model (Figure 1A) and treated it with 200 mg/kg and 400 mg/kg bw of APE daily. The results showed that APE significantly protected against *C. difficile*-induced colitis in mice (Figure 1D). Compared with the NC group, mice after *C. difficile* attack exhibited significant symptoms of infection, including weight loss, arching of the back, and wet tail. In contrast, APE attenuated the clinical signs in infected mice, which could be ameliorated by changes induced by colitis, including weight loss (Figure 1B), diarrhea, morbidity, and mortality (Figure 1C). Meanwhile, the HAP group showed less mortality than the LAP group. Histopathological examination was consistent with these findings, with reduced colonic crypt cells, thinning of the lamina propria, and unhealthy villi in the CDI group. The results of the H&E analysis of colon sections after APE intervention showed a significant reduction in tissue damage and inflammation, with more intact villi and less structural damage after polyphenol treatment (Figure 1E). H&E staining showed that APE prevented intestinal inflammation and that colon pathology scores were alleviated after APE treatment compared with the CDI group (Figure 1E, Supplementary Figure S1).

### 3.2. Effects of APE on Microbiota Community Composition

**Figure 2:**
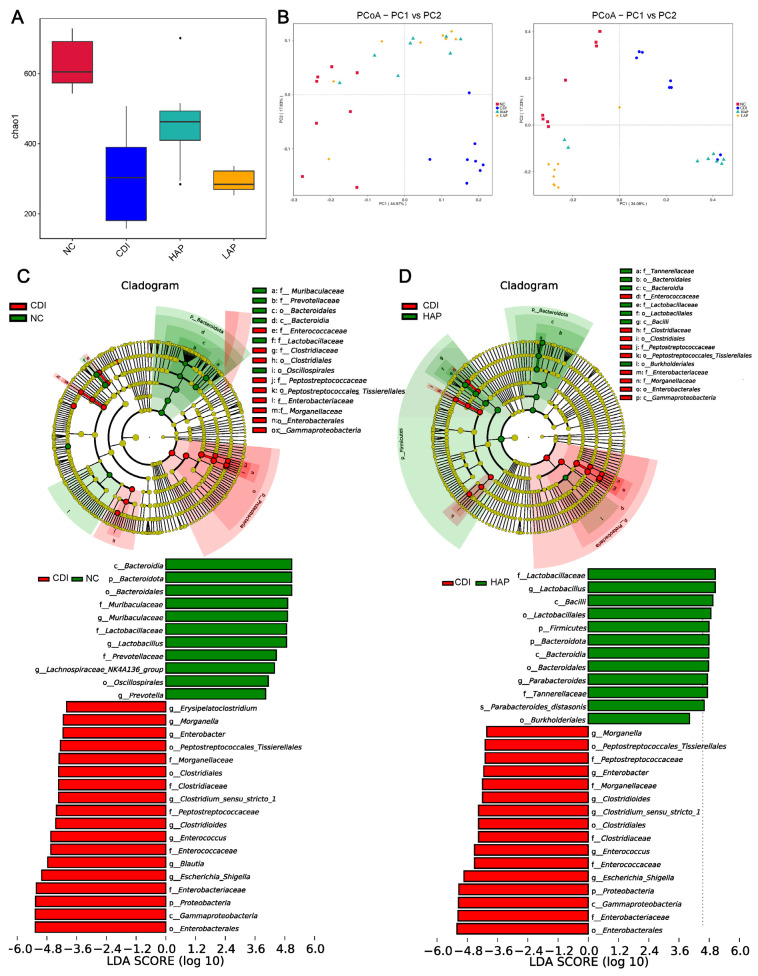
APE intervention changed the structure of intestinal microbiota. (**A**) Chao1 index. (**B**) PCoA plots based on weighted UniFrac (**left**) and unweighted UniFrac (**right**). (**C**) LEfSe branching plot between NC and CDI groups. (**D**) LEfSe branching plot between CDI and HAP groups. Data are expressed as mean ± SEM.

16S rRNA sequencing of the intestinal microbiota was carried out to analyze the bacterial community composition and to analyze the changes in intestinal microbiota associated with *C. difficile*-induced infection and the effects of APE on intestinal microbiota. To assess alpha diversity, lower microbiota richness (Chao1) was observed in the CDI group with respect to the healthy mice (Figure 2A). These results suggest that infection reduced the microbiota richness. After APE treatment, comparing the HAP and LAP groups with the CDI group, the Chao1 index of the HAP group increased (α-diversity), which showed that the HAP group increased richness. Weighted UniFrac and unweighted UniFrac were used to assess the overall microbiome composition (Figure 2B). The principal component analysis (PCoA) plot results showed that the microbial communities after APE treatment were significantly different from those in the NC and CDI groups.

To further explore the key bacteria associated with *C. difficile* infection and APE treatment, we performed linear discriminant analysis effect size (LEfSe) analysis. The CDI group was enriched in *Enterobacteriaceae*, *Peptostreptococcaceae*, and *Enterococcaceae* compared with the NC group (Figure 2C). In addition, infection reduced the abundance of *Muribaculaceae*, *Lactobacillaceae*, and *Prevotellaceae*. However, after the intervention with APE, the reduction in *Bacteroidota* and the increase in *Proteobacteria* were partially reversed (Figure 2D, Supplementary Figure S2). At the same time, APE increased the levels of *Lactobacillaceae*. *Lactobacillaceae* was associated with improved intestinal inflammation. Figure S3 showed the comparison of the taxonomic abundance (phylum, family, and genus). Similar to the LEfSe results, the APE treatment regulates the abundance of beneficial and harmful intestinal microbiota. The results suggest that intervention with apple polyphenols can mitigate the microflora disorder caused by *C. difficile* infection.

### 3.3. APE Protects the Integrity and Function of the Intestinal Barrier

**Figure 3:**
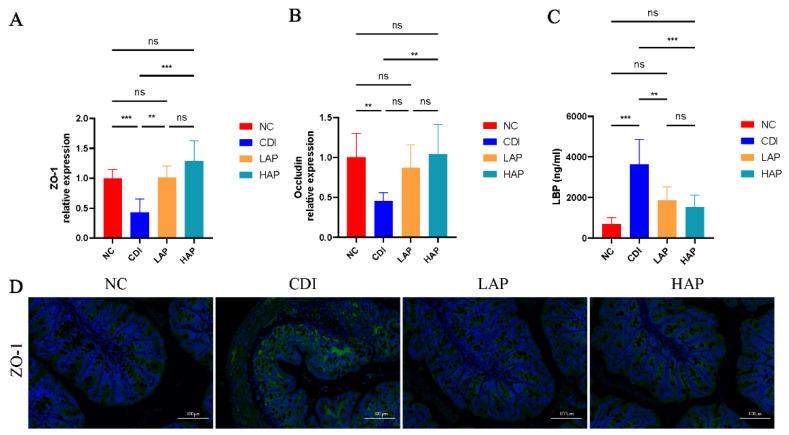
APE protects intestinal barrier function. (**A**,**B**) Relative mRNA expression of ZO-1 and occludin in colon tissue. (**C**) Serum LBP levels. (**D**) Representative immunofluorescence staining of ZO-1 in the colon. Data are expressed as mean ± SEM. ** *p* < 0.01, *** *p* < 0.001.

*C. difficile* infection disrupts the intestinal barrier [26] and increases intestinal permeability, so we further evaluated the benefits of APE on the intestine. We detected the colon mRNA expression (ZO-1 and occludin) to assess the intestinal barrier function. The barrier of the infected intestine was damaged, as evidenced by the reduced expression of ZO-1 and occludin (Figure 3A,B). This was improved by APE intervention, which showed an increase in ZO-1 and occludin. Meanwhile, we further analyzed the ZO-1 expression by immunofluorescence analysis (Figure 3D), and the results were consistent with the trend of mRNA levels. LBP is a marker of LPS [27], so we also measured the serum LBP level. The results showed that the LBP level after apple polyphenol intervention was reduced (Figure 3C), and APE may improve the upregulation of LPS levels induced by *C. difficile* infection. Therefore, the damage to the intestinal barrier caused by the *C. difficile* toxin was protected by APE intervention.

### 3.4. APE Attenuates C. difficile-Induced Systemic and Local Inflammatory Responses

**Figure 4:**
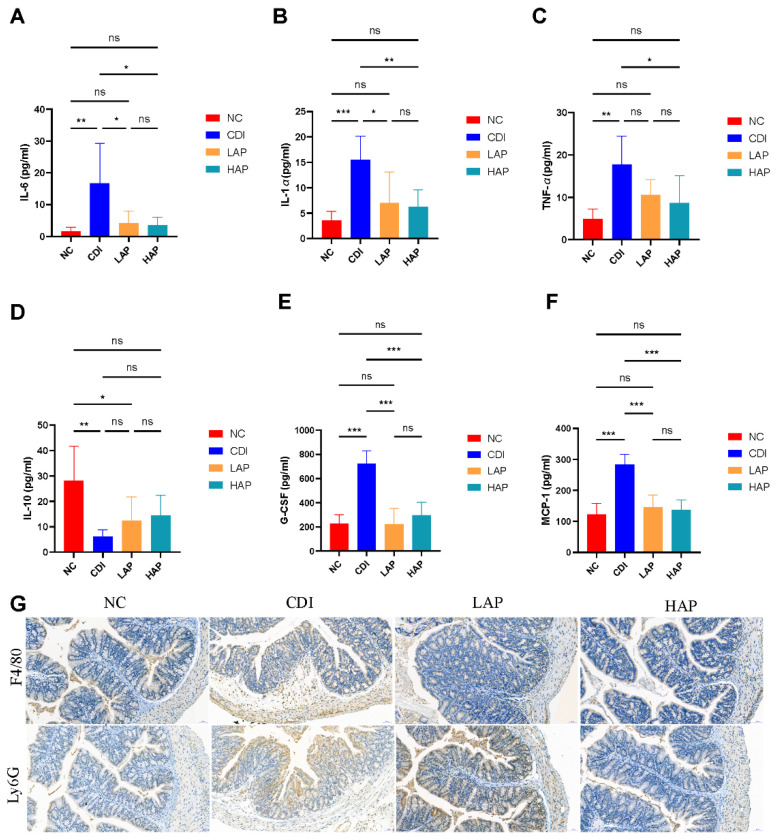
APE attenuated *C. difficile*-induced inflammatory. (**A**–**F**) Expression of cytokines (IL-6, IL-1α, TNF-α, IL-10, G-CSF, and MCP-1) in serum. (**G**) Immunohistochemical staining of colonic tissues for F4/80 and Ly6G indices. Data are expressed as mean ± SEM. * *p* < 0.05, ** *p* < 0.01, *** *p* < 0.001.

The *C. difficile* toxin induces an inflammatory response in the organism, leading to cytokine expression [28]. Our results showed that serum concentrations of inflammatory cytokines (Figure 4A–F), such as IL-6, IL-1α, TNF-α, IL-10, G-CSF, and MCP-1, were upregulated in the CDI group with respect to the NC group. After APE intervention, the levels of cytokines in the LAP and HAP groups declined, whereas the high-dose APE group showed better efficacy than the low-dose group. Cytokines are closely related to inflammatory cells, and toxins lead to the aggregation of macrophages and neutrophils in the colon [29]. Furthermore, we evaluated macrophages (F4/80+) and neutrophils (Ly6G+) in colon tissue (Figure 4G), and immunohistochemistry displayed that the CDI group was infiltrated with macrophages and neutrophils, which was ameliorated by APE.

### 3.5. Effects of APE on C. difficile-Induced Metabolic Disorders

**Figure 5:**
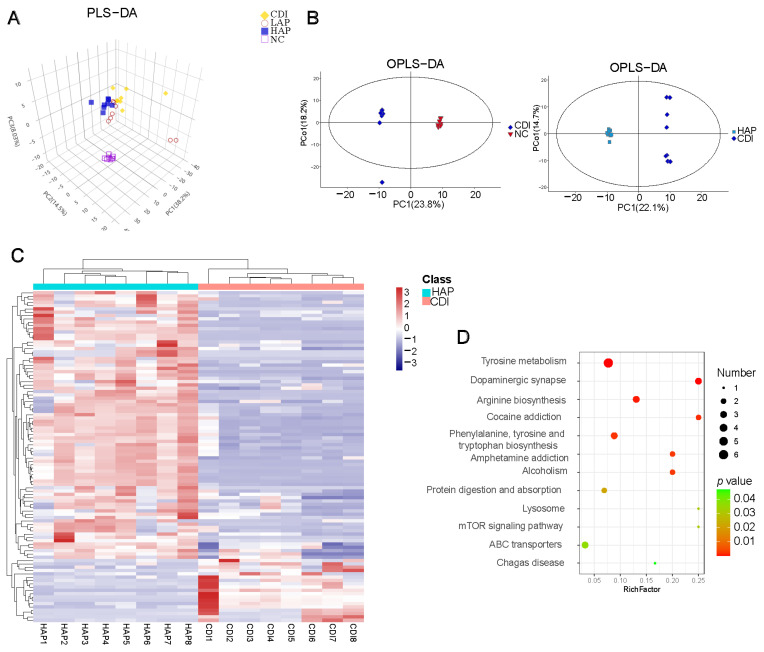
APE attenuated *C. difficile*-induced metabolic disorders. (**A**) PLS-DA shows metabolic structures of different groups. (**B**) OPLS-DA plot comparing groups. (**C**) Heatmap of differential metabolites between HAP and CDI groups. (**D**) KEGG pathway enrichment map. Circle size shows the number of enriched metabolites, and color scale represents *p* value.

To determine the effects of APE on the intestinal metabolome, we analyzed cecum contents using GC-MS. A partial least squares–discriminant analysis (PLS-DA) plot showed that the LAP, HAP, CDI, and NC groups were significantly separated in metabolism (Figure 5A), presenting the metabolic differences. An orthogonal PLS-DA (OPLS-DA) plot presented the different profiles between groups (Figure 5B and Supplementary Figure S4). Based on the metrics, variable importance in the projection (VIP), fold changes, and *p* values, we screened for differential metabolites between the CDI and HAP groups (Figure 5C, Supplementary Figure S5), which showed that 81 metabolites were significantly upregulated and 19 metabolites were downregulated in the HAP group compared with the CDI group. The heatmap (Figure 5C) shows the differential metabolites involved in pathways including lipid, amino acid, and carbohydrate metabolism. Moreover, Figure S6 shows the heatmap of metabolites among the four groups. KEGG pathway enrichment analysis screened the CDI and HAP groups for major metabolic pathways (Figure 5D), including tyrosine metabolism, arginine biosynthesis, phenylalanine, tyrosine and tryptophan biosynthesis, protein digestion and absorption, and the mTOR signaling pathway. L-arginine, a metabolite related to arginine metabolism, was significantly increased in the HAP group. L-tryptophan and phenylacetic acid were decreased in the HAP group.

We also quantified the SCFA levels in the cecum of mice (Supplementary Figure S5). As shown in Figure S7, the concentrations of SCFAs in the NC and CDI groups were significantly different between acetic acid, butyric acid, propionic acid, and valeric acid. Oral administration of APE significantly increased the levels of SCFAs compared with the CDI group.

### 3.6. APE Protects against Changes in Colon Transcriptional Regulation

**Figure 6:**
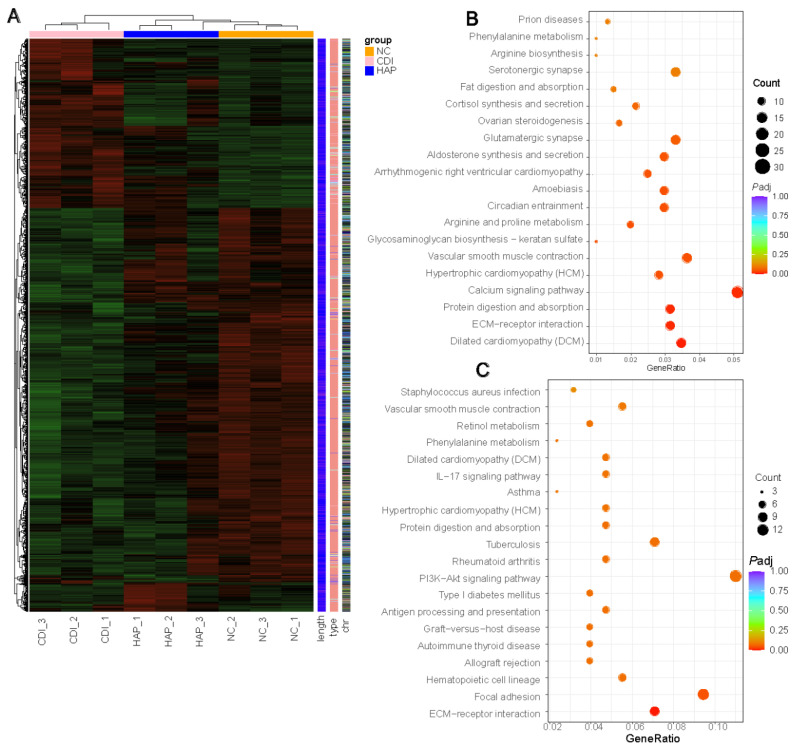
Partial reversal of transcriptional regulatory changes by apple polyphenol treatment. (**A**) Heatmap of differentially expressed genes among the 3 groups. (**B**) KEGG map of differentially expressed genes between CDI and NC groups. (**C**) KEGG map of differentially expressed genes between CDI and HAP groups. Circle size shows number of genes enriched in the pathway, and color scale represents *p* value.

Furthermore, the gut microbiome regulates host immune defenses in addition to colonization resistance. Transcriptome sequencing (RNA-seq) analysis was performed on colon tissue to determine which networks may be associated with the benefits of polyphenols. Deep sequencing was applied to determine the overall expression differences between the control and treated groups (NC = 3, CDI = 3, HAP = 3). Using the criteria of |log2 (fold change) |> 1 and *P* _adj_ < 0.05, we analyzed genes that significantly differed both between the NC and CDI groups and between the CDI and HAP groups to analyze the genes in the colon tissue that were altered by *C. difficile* infection but mitigated by APE (Figure 6A). First, apple polyphenols attenuated the *C. difficile*-induced upregulation of 94 gene transcripts in the colon tissue, including chemokines for neutrophil and monocyte recruitment (*Cxcl5*, *Cxcl1*, *Cxcl13*), inflammation-related genes (*Ly6a*, *Ly6c1*, *Lbp*, *Lcn2*), immunomodulation (*Ighv1-54*, *Cd14*), and oxidative stress (*Hif1a*, *Hif3a*, *Hspa12a*). Second, the downregulation of *C. difficile*-induced transcription of 157 genes was also attenuated with apple polyphenols, including lipid metabolism (*Apol7c*, *Hsd3b2*, *Hsd3b3*), intestinal barrier function (*Muc2*, *Muc3a*, *Ms4a7*), and immune response (*Ccl5*, *Cd3g*, *Cd74*, *Ighv10-3*, *Ighv1-20*, *Ighv5-6*, *Igkv4-57*, *Igkv5-39*, *Igkv8-19*). The results of the pathway enrichment analysis of differentially expressed genes using KEGG revealed differential pathways between the CDI and NC groups, including extracellular matrix (ECM)–receptor interaction, protein digestion and absorption, calcium signaling pathway, glycosaminoglycan biosynthesis-keratan sulfate, and arginine and proline metabolism (Figure 6B). Differential pathways between the HAP and CDI groups included focal adhesion and ECM–receptor interaction (Figure 6C).

## 4. Discussion

The concept of health promotion and disease prevention is important. *C. difficile* infection threatens the human health, and intestinal microbiota disorders are associated with the occurrence of CDI [30]. Through the gastrointestinal tract, unmodified polyphenols (90–95%) accumulate at high concentrations in the colon and are then degraded by the intestinal microbiota into numerous lower molecular compounds [31]. It is therefore worthwhile to investigate how dietary polyphenols can modulate the intestinal microbiota and metabolism to prevent and treat disease. In our study, gut microbiota and metabolism exert the beneficial effects of APE. Furthermore, APE treatment increased intestinal microbiota abundance in CDI mice, with a significant increase in the relative abundance of *Lactobacillaceae* and a lower relative abundance of *Enterococcus* and *Enterobacteriaceae*. The mechanism by which APE ameliorates infection is associated with the regulation of immune, infection, and metabolic pathways.

Our study showed that APE can restore the dysbiosis of intestinal microbiota caused by *C. difficile*. Polyphenols can be biotransformed into metabolites in the intestine and promote the number of beneficial bacteria and relatively reduce the growth of pathogenic bacteria [32]. Therefore, based on the improvement of intestinal inflammation by APE, it is reasonable to speculate that APE exerts its metabolic functions mainly through the regulation of intestinal microbiota and their metabolites. *C. difficile* infection led to a decline in microbial diversity, and APE increased the richness. In general, the ratio of *Proteobacteria* to *Bacteroidota* reflects the gut microbiota balance and its relationship to inflammation [33]. APE significantly reduced the increase in the ratio of *Proteobacteria* to *Bacteroidota* caused by CDI, suggesting that APE may suppress inflammation by maintaining intestinal microbiota homeostasis. In addition, *Bacteroidota* could provide most of the acetate and propionate.

In addition, previous pieces of evidence support the role of SCFAs as key molecules mediating the interaction between microbial and host metabolism, and SCFA-producing bacteria (such as *Muribaculaceae* and *Lactobacillaceae*) have attracted the attention of researchers [34,35]. Our study showed that some of the bacteria causing microbiome changes were *Lactobacillaceae*, which were substantially reduced after antibiotic treatment, suggesting the ability of strong suppressors for *C. difficile* [36]. Various intestinal microorganisms promote the metabolism transformation of the indigestible carbohydrates into different SCFAs. The chemical properties of SCFAs are well-documented, and their health effects have been widely demonstrated [37]. These substances can act through a variety of metabolic pathways in the intestine and distant sites, such as the muscle, liver, and brain [38]. In addition, butyrate also improves the intestinal barrier through hypoxia-inducible factor 1 (HIF-1), whose overexpression improves the barrier function and reduces inflammation in intestine, thus providing protection against *C. difficile* [39]. In particular, *Lactobacillaceae* are a major bacterial family in many mammalian gut microbial communities, and the host receives benefits from many members of this family [40]. Members of this family are also resistant to colonization by *C. difficile* [41], which is an inhibitor of *C. difficile* growth. Thus, *Lactobacillaceae* appear to fight *C. difficile* through independent mechanisms, including resource competition and inhibitory SCFA and secondary bile acid production [42]. In our study, infection resulted in an increase in oligopeptides and free amino acids associated with *C. difficile* germination in the canal lumen and, when combined with the loss of *Lactobacillaceae*, had a selective advantage over *C. difficile*.

The microbiota and its metabolites exist close to the intestinal epithelium, which separates the host from the outside. The epithelial barrier is formed by tight junctions, such as claudins, ZO-1, and occludin [43]. Our results show that APE ameliorates colonic pathological damage, protects the intestinal barrier, and suppresses the intense cytokine inflammatory response in infected mice. In addition to blocking inflammation, intestinal permeability is prevented by certain microbial metabolites that enhance the barrier function. It would be advantageous to treat CDI with such microbial metabolites. Therefore, the consumption of a polyphenol-rich diet is necessary but not sufficient for their health effects; microorganisms that convert polyphenols into beneficial metabolites must also be present. Physiological processes are largely unknown to the targets or pathways of these microbial metabolites.

Disturbances in intestinal metabolism are closely associated with the progression of *C. difficile* infection. For example, various amino acids and primary bile acids promote spore germination, but secondary bile acids inhibit spore germination [44]; fermentation of amino acids or carbohydrates affects growth [45]. Metabolites produced by the gut microbiota can make close communication with the organism as well [46]. Our results showed that disordered metabolites such as L-arginine were improved by APE intervention, and pathway enrichment showed improvement in metabolic pathways such as arginine biosynthesis. Another study showed that the expression of arginine and ornithine metabolic pathways is increased in mice resistant to *C. difficile* infection [47]. Cecum contents also indicated a positive modulatory effect of APE on *C. difficile* infection.

Microorganisms and the host immune system actively interact in the intestine, and the gut microbiota plays a key role in establishing immune homeostasis [48]. We explored the mechanism of APE protection against *C. difficile* infection by transcriptomics. In contrast to our serologic and pathologic results showing improvement in inflammation and immune damage after APE treatment, the transcriptomic results showed that polyphenols modulated inflammation-related genes in the organism after intervention. *C. difficile* infection caused alterations in pathways such as immune system pathways and cellular signal transduction pathways [49]. The improvement of these pathways after APE treatment suggests that immunomodulatory mechanisms of APE influence alterant metabolites, immunity, and maintenance of physiological activities in infected mice.

Our experiment has some shortcomings. We did not collect feces before the antibiotic treatment and analyze the microbiota structure before and after modeling, which will be remedied in later experiments.

## 5. Conclusions

Our study examined the therapeutic effects and mechanisms of APE on CDI. A positive therapeutic effect of APE on CDI was demonstrated in our study, and CDI mice treated with APE exhibited delayed disease progression and a significant reduction in disease severity. In addition, APE treatment significantly improved the structural and metabolic changes in the disordered intestinal microbiota. The data indicate that APE is effective in reducing CDI clinical signs, inhibiting proinflammatory cytokines, and increasing anti-inflammatory cytokines by altering the intestinal microbiota. More efforts are needed to develop a theoretical framework for the exploitation of apple resources in-depth based on our findings.

## Data Availability

Not applicable.

## Supplementary Materials

The following supporting information can be downloaded at: https://www.mdpi.com/article/10.3390/metabo12111042/s1, Figure S1: H&E scores of colonic tissues of mice in each group, Figure S2: Linear discriminant analysis (LDA) effect size (LEfSe) analysis among LAP and CDI groups, Figure S3: Relative abundance of abundant taxa at phylum, family, and genus level, Figure S4: OPLS-DA plot showing metabolic profiles of CDI and LAP groups, Figure S5: Heatmap of differential metabolites between HAP and CDI groups, Figure S6: Heatmap of differential metabolites between groups, Figure S7: Levels of SCFAs between different groups; Table S1: PCR primers.
