# Supplementary material for: Apple Polyphenol Extract Suppresses Clostridioides difficile Infection in a Mouse Model
Source: Metabolites. 2022 Oct 30;12(11):1042. doi: 10.3390/metabo12111042 (PMC9694464; doi:10.3390/metabo12111042)

Supplemental Material

Supplementary Table. S1. PCR primers.

Gene	Forward Sequence (5'-3')	Reverse Sequence (5'-3')
Bacteria16S rRNA V3V4	TACGGRAGGCAGCAG	AGGGTATCTAATCCT
β-actin	AGTGTGACGTTGACATCCGT	GCAGCTCAGTAACAGTCCGC
Occludin	TTCCTCTGACCTTGAGTGTGG	CTCTTGCCCTTTCCTGCTTT
ZO-1	GCCGCTAAGAGCACAGCAA	GCCCTCCTTTTAACACATCAGA

Supplementary Figure S1. H&E scores of colonic tissues of mice in each group.

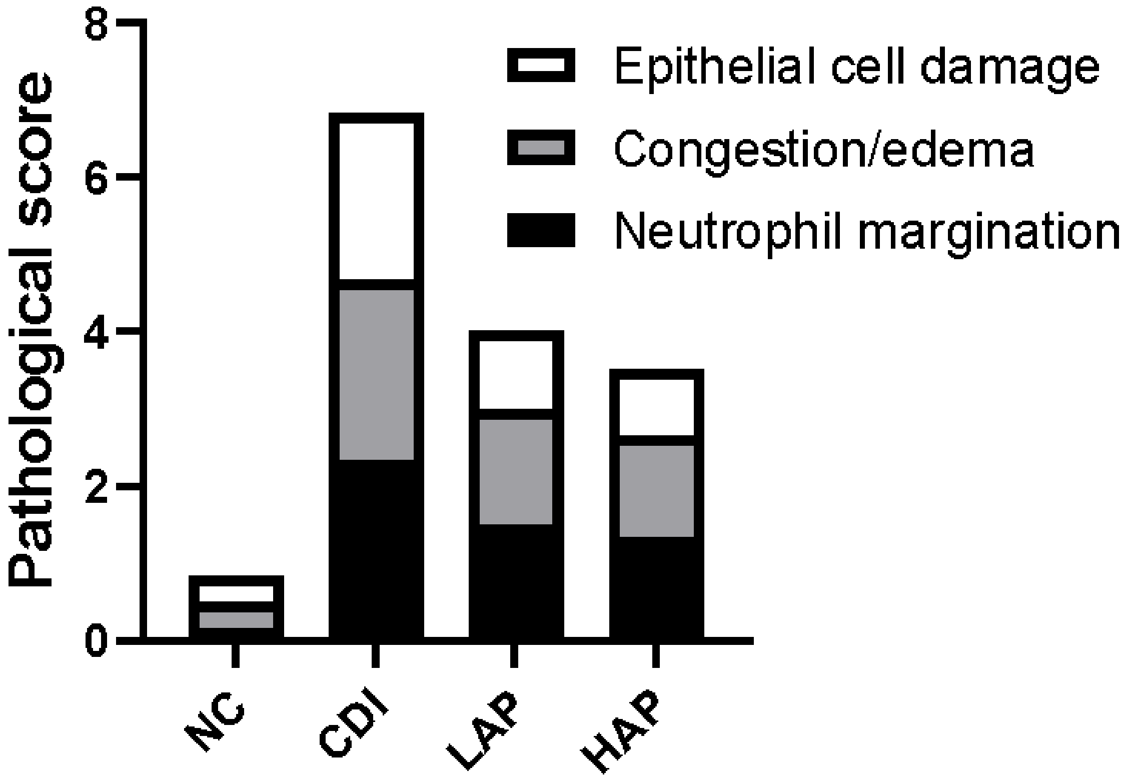

Supplementary Figure S2. Linear discriminant analysis (LDA) effectsize (LEfSe) analysis among LAP and CDI group.

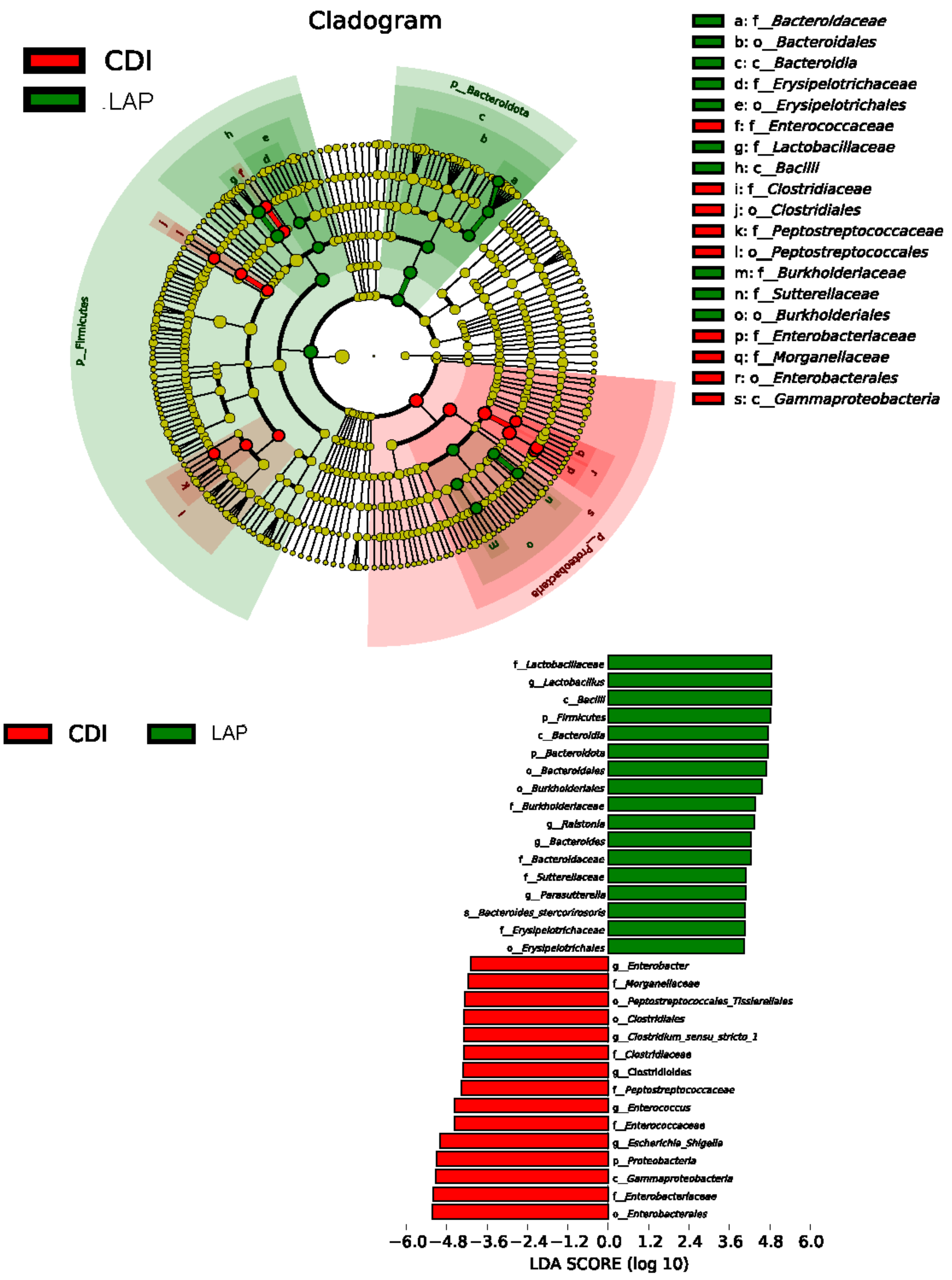

Supplementary Figure S3. Relative abundance of abundant taxa at phylum, family, and genus level.

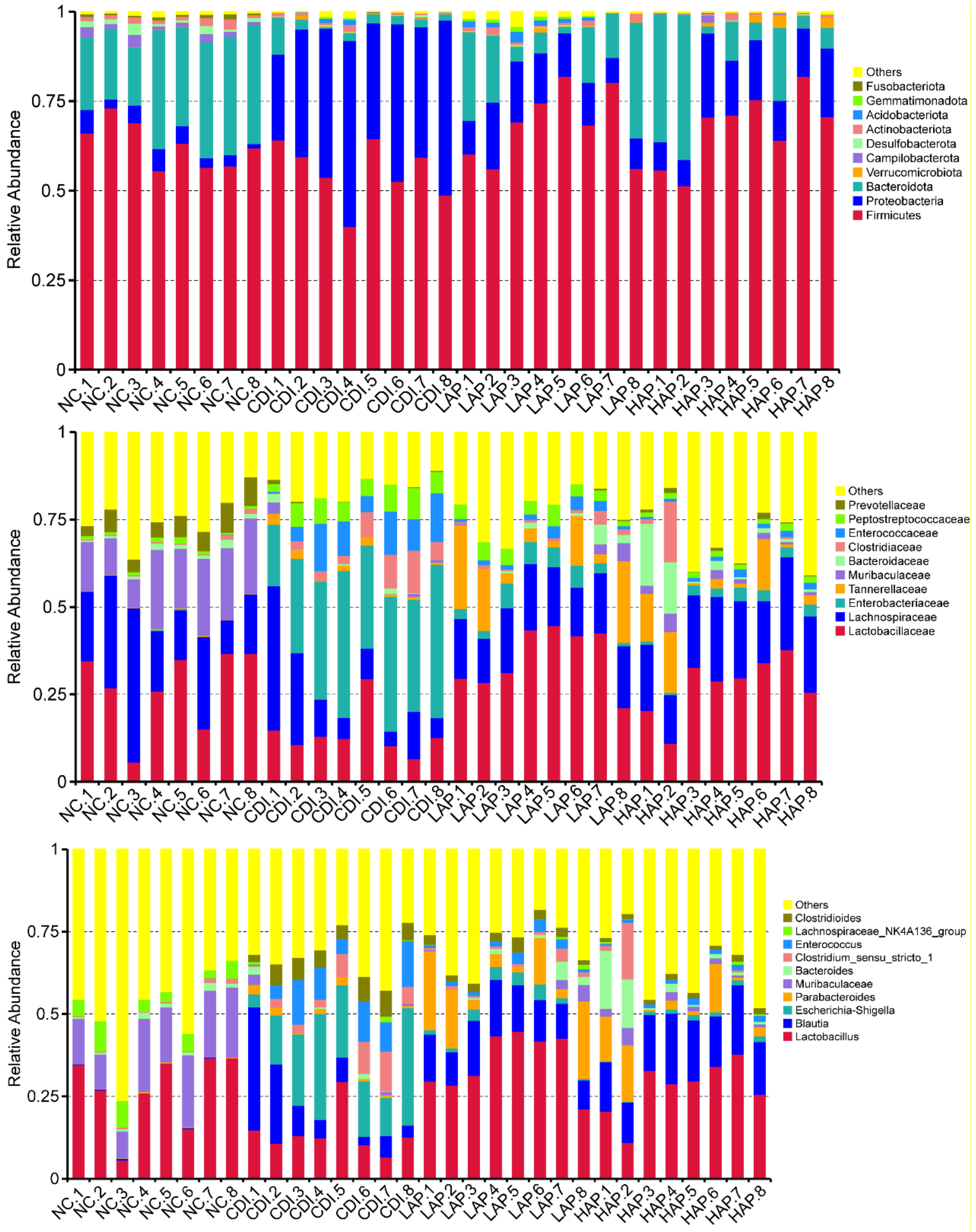

Supplementary Figure S4. OPLS-DA plot showing the metabolic profiles of the CDI and LAP groups.

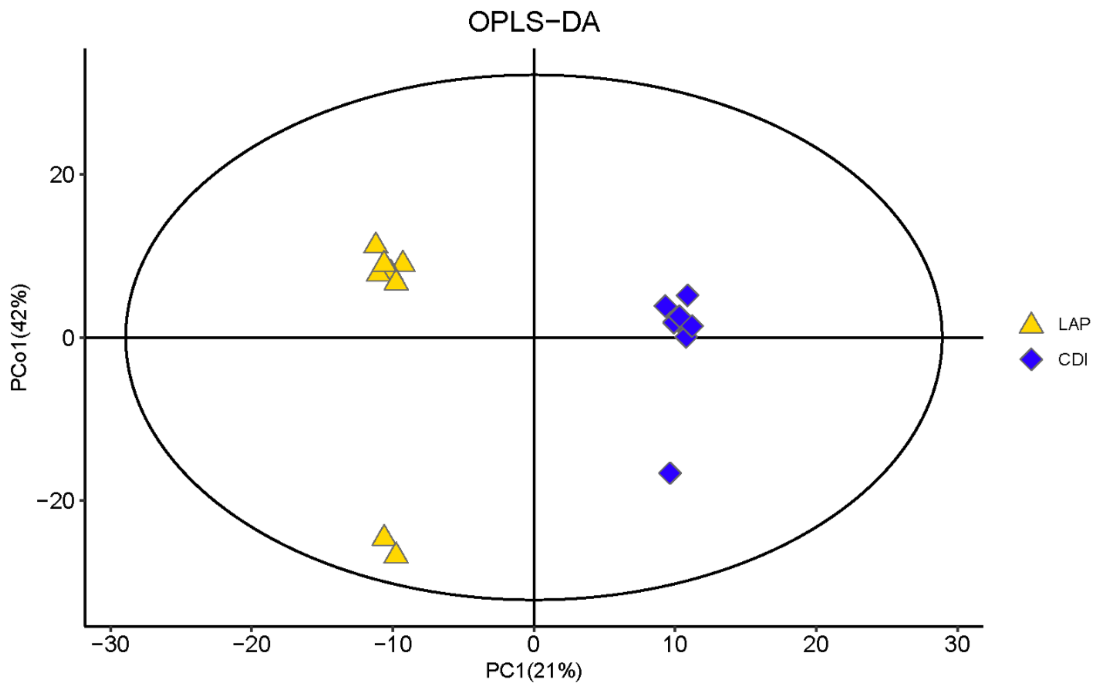

Supplementary Figure S5. Heatmap of differential metabolites between the HAP and CDI groups.

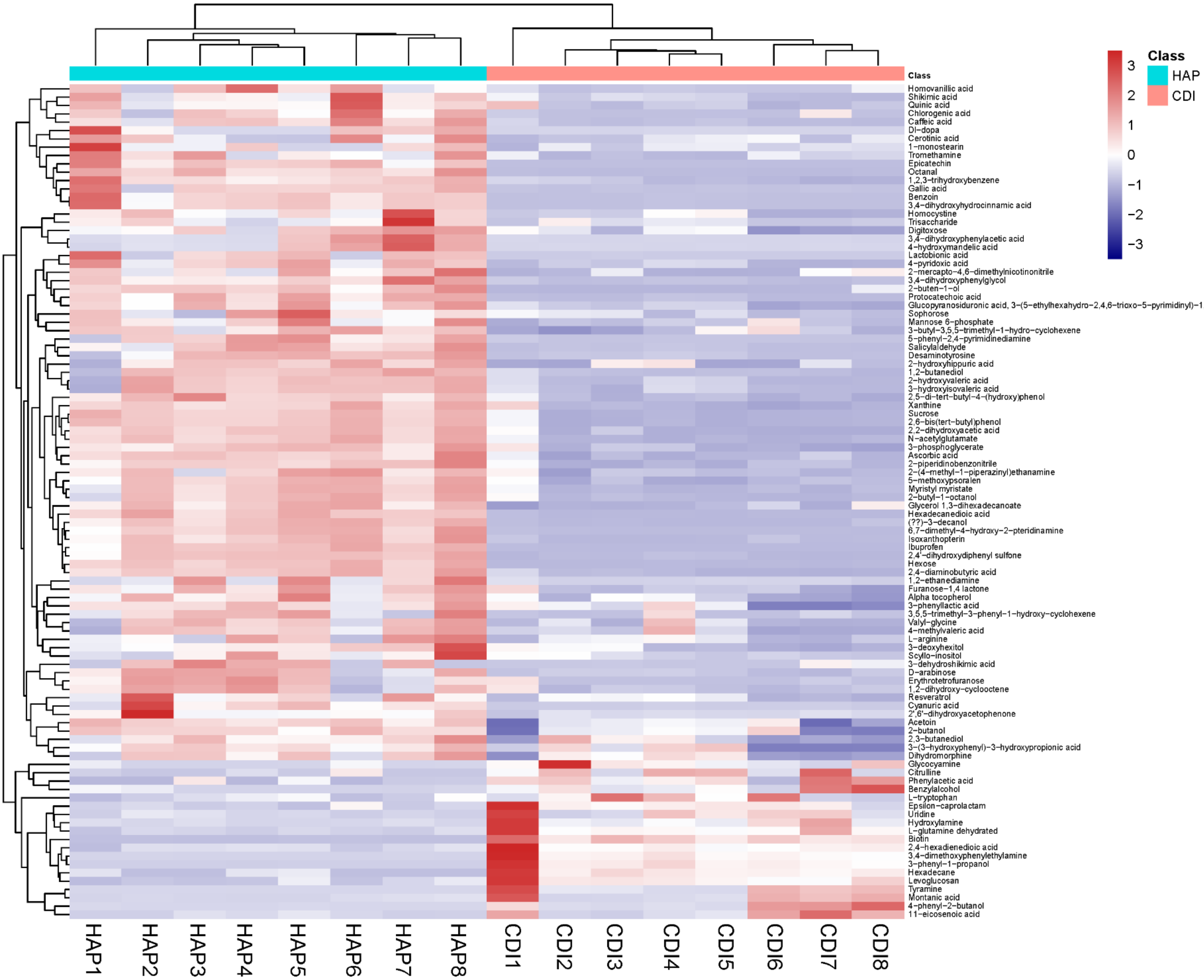

Supplementary Figure S6. Heatmap of differential metabolites between groups.

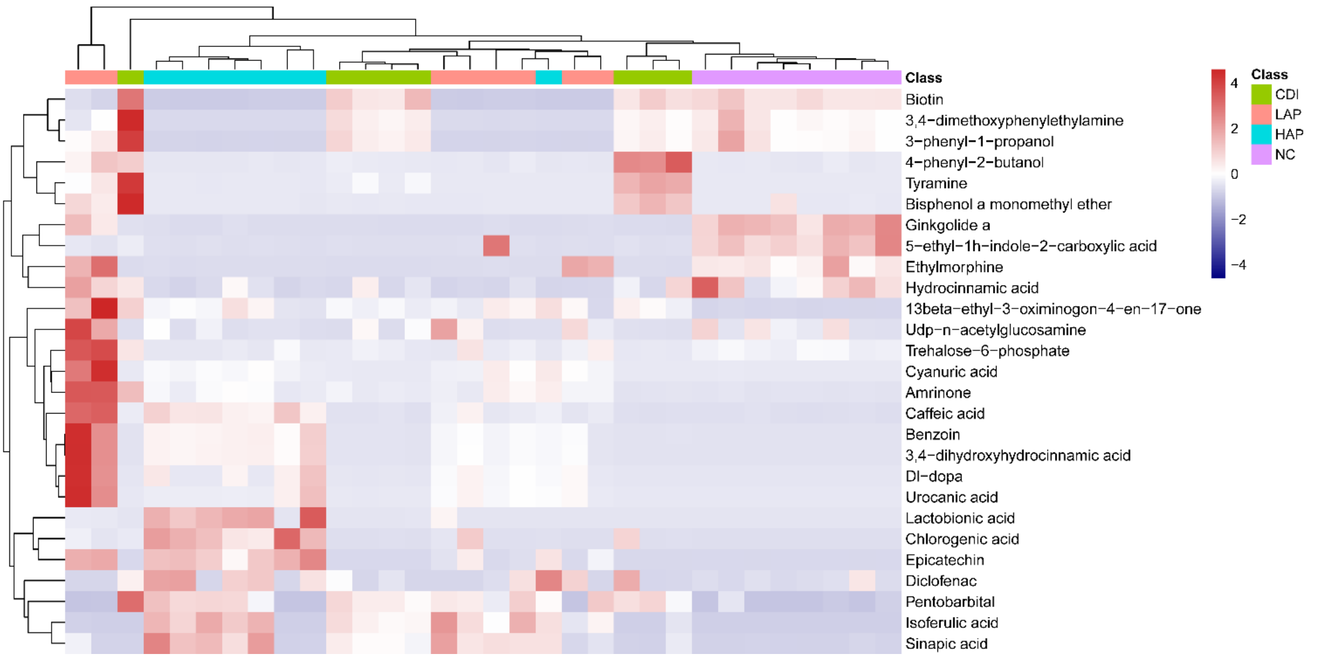

Supplementary Figure S7. The levels of SCFAs between different groups. The data are expressed as the mean ± SEM. *, *P* < 0.05; **, *P* < 0.01; ***, *P* < 0.001; and ****, *P* < 0.0001.

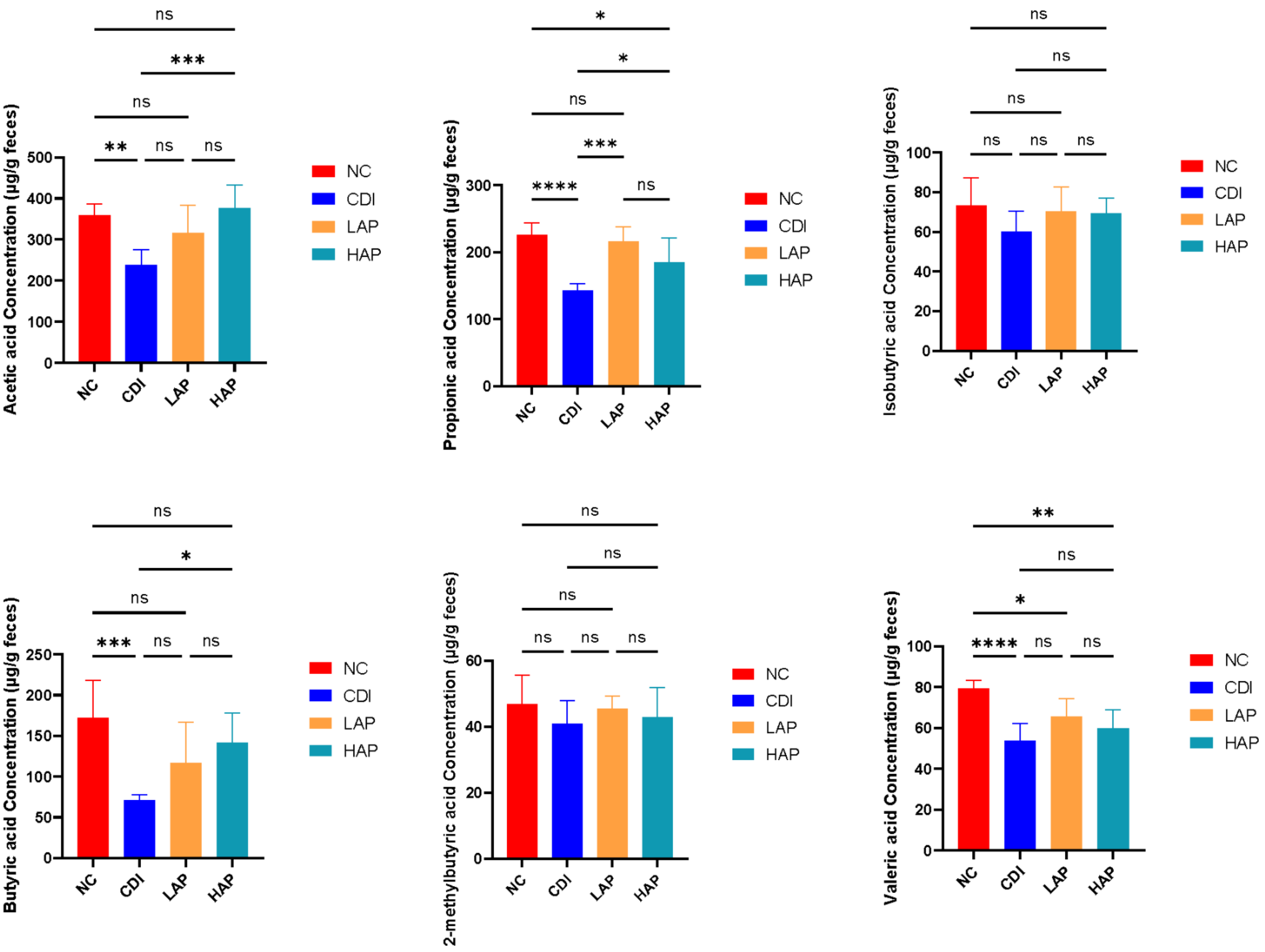

Supplement: Supplementary file 1 [file metabolites-12-01042-s001.zip › metabolites-1969569-supplementary.pdf]
